# Screen in between: How does livestreaming impact patient participation in education?

**DOI:** 10.1111/medu.15585

**Published:** 2024-11-27

**Authors:** Kelvin Gomez, Jane Kirby

**Affiliations:** ^1^ Leeds Institute of Medical Education, Leeds School of Medicine University of Leeds Leeds UK; ^2^ Leeds Institute of Health Sciences, Leeds School of Medicine University of Leeds Leeds UK

## Abstract

Gomez and Kirby suggest that while livestreamed clinical experiences offer potential solutions to placement challenges, careful design is needed to ensure meaningful patient involvement and address risks like diminished rapport and anxiety.

The Hippocratic Corpus—a collection of ancient Greek medical texts from 420 and 370 BC—are among the earliest recorded evidence available on medical theory and practice.^1^ Centuries later, inspired by Hippocratic medicine, Galen of Pergamum, a prominent Greek physician of his time, set about advancing thinking in medical theory and practice. His influence led to a division between the two, with the former considered a more notable pursuit. This led to the emergence of ‘theoreticians’ who prioritised knowledge over practice and who became recognised as the ‘true’ physicians.^1(p.64^ ^)^

We start with this history not as a means to advocate for ancient medical thinking in our curricula, but to induce reflection on the variety of challenges medicine has faced over the years. For physicians in Ancient Greece, the challenge was a lack of knowledge and understanding. Fast track to the 21st century, and we find ourselves seemingly at the opposite end of the spectrum, where significant advancements in understanding of diverse diseases and treatments contribute to overcrowding medical school curricula and consequent impacts on learning.^2^

Rather than sufficing to be ‘theoreticians’, however, medical students of today are expected to gain sufficient clinical exposure to enable good practice while learning from an increasing diversity of patients and medical cases. Doing so has traditionally required in‐person access to such experiences, creating considerable challenge to institutions and placement providers as the content to be learned and student numbers expand. A second, and no less critical, aspect of enabling sufficient practice derives from global healthcare workforce shortages and maldistribution exacerbating the challenge by encouraging (or requiring) training in more remote environments where there can be less opportunity to encounter the full range of patients. Our recent work has highlighted that livestreaming clinical experiences (LCEs) between a patient and a clinical educator to remotely‐located students may be one potential solution to this problem,^3^ although it too has limitations. Despite potential challenges, we owe it to the healthcare education community to explore technological solutions that open up opportunities to help support the workforce crisis.

A combination of recent developments in digitilisation and shifts in perceptions towards remote consultations, due to the Covid‐19 pandemic, have made LCEs more acceptable as a form of clinical learning. However, such shifts to online learning do change the fundamental triadic interactions between patients, students and clinicians. This has prompted us to consider how this shift may impact patient involvement during online clinical experiences. With this in mind, we read the manuscript by Bennett‐Weston and Gay^4^ on patient involvement in healthcare education with particular interest. How might patient involvement during healthcare education be impacted—for better or worse—when engaging in LCEs?

Centred on the ‘Spectrum of Involvement’—a framework that defines hierarchical levels of patient involvement—Weston and Gay's paper uses a case study approach to understand how patients, students and educators experience and view patient partnerships in healthcare education. The findings challenge the idea that equal patient partnerships should be the only desirable objective in healthcare education. Instead of maximal patient involvement, the authors argue in favour of ensuring patients feel valued, irrespective of their level of involvement. Respect, remuneration and meaningful engagement are anticipated to impact patients' sense of feeling valued when involved in healthcare education.

Interestingly, our recent study on patient perceptions of LCEs in medical education has highlighted that there is a tendency for patients to forget that students are present in the consultation room despite being able to hear them or see them on a screen.^5^ This suggests that without thoughtful design of LCEs that seek to actively involve patients, there may be a risk of diminishing patients' sense of involvement in healthcare education during LCEs. In turn, there may be a risk of declines in other reported benefits, such as the increase in self‐esteem patients report when engaging in healthcare education.^6^

There may be a risk of diminishing patients' sense of involvement in healthcare education during LCEs.

The impact of technology‐mediated interaction such as that required for LCEs also warrants discussion.^7^ The two‐dimensional projection of an individual on a screen often results in a reduction of non‐verbal cues due to the cropped visual.^7^ General patterns of interaction in an online setting are also different. Take a simple farewell when concluding a video call as an example—I continue to be guilty of waving goodbye to colleagues despite never doing the same during in‐person interactions. From a patient's point of view, I can only imagine that such interactions may feel less ‘human‐like’, especially if the ability to see students is impacted by the size or position of a screen. This may have further implications on patient anxieties that have been reported to arise when participating in healthcare education; feeling judged by students as well as consent and confidentiality concerns may be heightened in an online context where learners are unknown to the patient and potentially invisible (depending on the number of the observers watching the clinical encounter).

Consent and confidentiality concerns may be heightened in an online context.

One could argue that a clear benefit of videoconferencing is its time efficiency, as meetings are typically more structured and tend to follow an agenda. Here again though, this perceived advantage seems to be at odds with the benefits patients have reported of having more time available for rapport‐building and companionship, particularly with students, when patients engage in education.^6^ A reasonable question to ask, therefore, is whether the lack of rapport‐building creates a barrier to communication that may impact patients' willingness to offer their body and authenticity in the future.^8^ This may, in turn, impact clinicians' willingness to teach students through these novel methods of delivery.

Creates a barrier to communication that may impact patients' willingness to offer their body and authenticity.

As always, the acceptability of risks must always be considered against the potential for rewards, especially if there is a chance that risk can be mitigated. The poor distribution of the workforce is widening global health inequalities. Doctors are migrating to more affluent areas in search of better working conditions in the hope of holding off burnout. Of relevance here is another article in this issue from Mizumoto et al.^9^ that highlights the need to counteract the inverse care and training law, that those with greater healthcare need receive less healthcare. Their study sets out to understand the factors that cultivate a passion for working with patients in complex and challenging social situations (CCSS). A central theme from their research is the joy derived from interacting with patients, going on a journey with them over time, understanding their personal lives and the impact this had on health and health outcomes. They highlight an educational opportunity and hope that this understanding will help cultivate a positive attitude towards the caring of patients in CCSS while leading to greater recruitment of physicians to these areas. From this perspective, LCEs may have a role in spreading the desired attitudes to a diverse population of students, offering opportunities not widely accessible through traditional clinical placements.

LCEs may have a role in spreading the desired attitudes to a diverse population of students.

In other words, rather than telling students about the potential benefits, we can use technology to show them. This approach may help close the inverse training law gap and offer patients from CCSS the chance to participate in student education, echoing the positive outcomes noted by Weston and Gay.^4^ However, challenges remain. Research suggests that simple exposure to inclusion health groups can sometimes reinforce misinformation and stigma.^10^ Educators must carefully navigate this balance between improving access and worsening stigma and misunderstanding. Could involving individuals with lived experience in the education encounter help mitigate this?

Educators must carefully navigate this balance between improving access and worsening stigma and misunderstanding.

From a traditional perspective, it is easy to view patient involvement in LCEs as simply a constricted version of in‐person clinical experience. Such narratives are often taken up by those who view technology as a ‘dehumanising force’ that seeks to substitute rather than enrich healthcare education.^11(p.11^ ^)^ Perhaps it is time to confront the idea that LCEs may simply be inherently different forms of patient participation with different sets of strengths and weaknesses, one of many methods of participation as described in Bennett‐Weston and Gay's Wheel of Patient Partnerships. Perhaps then we could determine how and when LCEs benefit the broader imperative of sustainable healthcare education through thoughtful implementation, rather than acting as though there is a simple universal ‘if’ they provide benefit that can be proven.

## AUTHOR CONTRIBUTIONS

**Kelvin Gomez:** Conceptualization; writing – original draft. **Jane Kirby:** Conceptualization; writing – original draft.

## CONFLICT OF INTEREST STATEMENT

No conflict of interest to declare.
